# Summary of taxonomy changes ratified by the International Committee on Taxonomy of Viruses (ICTV) from the Archaeal Viruses Subcommittee, 2025

**DOI:** 10.1099/jgv.0.002117

**Published:** 2025-07-25

**Authors:** Mart Krupovic, Diana P. Baquero, Eduardo A. Bignon, Ariane Bize, Guillaume Borrel, Mingwei Cai, Lanming Chen, Marion Coves, Changhai Duan, Simonetta Gribaldo, Eugene V. Koonin, Meng Li, Lirui Liu, Yang Liu, Ying Liu, Sofia Medvedeva, Yimin Ni, Apoorva Prabhu, Christian Rinke, Yongjie Wang, Tianqi Xu, Shuling Yan, Qinglu Zeng, Rui Zhang, ICTV Taxonomy Summary Consortium, E.M. Adriaenssens

**Affiliations:** 1 Institut Pasteur, Université Paris Cité, CNRS UMR6047, Archaeal Virology Unit, Paris, France; 2 Université Paris-Saclay, INRAE, PROSE, Antony, France; 3 Institut Pasteur, Université Paris Cité, CNRS UMR6047, Evolutionary Biology of the Microbial Cell, Paris, France; 4 Institute for Advanced Study, Shenzhen University, Shenzhen, PR China; 5 College of Food Science and Technology, Shanghai Ocean University, Shanghai, PR China; 6 The Hong Kong University of Science and Technology, Hong Kong, PR China; 7 Computational Biology Branch, Division of Intramural Research, National Library of Medicine, National Institutes of Health, Bethesda, MD, USA; 8 Australian Centre for Ecogenomics, The University of Queensland, Brisbane, Australia; 9 University of Innsbruck, Innsbruck, Austria; 10 Entwicklungsgenetik und Zellbiologie der Tiere, Philipps-Universität Marburg, Marburg, Germany; 11 Department of Ocean Science, The Hong Kong University of Science and Technology, Hong Kong, PR China

**Keywords:** *Adrikavirales*, *Ageovirales*, *Agnivirus*, *Agnivirus brisbanense*, *Apasviridae*, *Aprofuvirus*, *Aprofuvirus guaymasense*, *Avenivirus*, *Avenivirus atlanticense*, *Baiafusivirus*, *Baiafusivirus chesapeakense*, *Baiafusivirus delawarense*, *Calorviricota*, *Caminiviricetes*, *Chiyouviridae*, *Dafengvirus*, *Dafengvirus linsing*, *Eurekaviridae*, *Fuxiviridae*, *Geogavirus*, *Geogavirus atlanticense*, *Geogavirus guaymasense*, *Geogavirus pacificense*, *Hesperidvirus*, *Hesperidvirus aureum*, *Hewusuvirus*, *Hewusuvirus methanobrevibacteri*, *Huangdiviridae*, *Krittikaviridae*, *Kunpengviridae*, *Manusuvirus*, *Manusuvirus methanobrevibacteri*, *Marefusivirus*, *Marefusivirus columbiaense*, *Marefusivirus helgoense*, *Marefusivirus jervisense*, *Marefusivirus montereyense*, *Marefusivirus pacificense*, *Nipumfusiviridae*, *Satyavativiridae*, *Taijivirus*, *Taijivirus yinyang*, *Terrafusivirus*, *Terrafusivirus michiganense*, *Terrafusivirus tennesseense*, *Thalassapleoviridae*, *Usuviridae*, *Velanvirus*, *Velanvirus brisbanense*, *Vyasavirus*, *Vyasavirus brisbanense*, *Wargodvirus*, *Wargodvirus xiongnu*, *Xuanyuanvirus*, *Xuanyuanvirus yandi*, *Yangshanfusivirus*, *Yangshanfusivirus mimetica*

## Abstract

An erratum of this article has been published full details can be found at 10.1099/jgv.0.002145

The International Committee on Taxonomy of Viruses (ICTV) holds a ratification vote annually following the review of newly proposed taxa by ICTV Study Groups and members of the virology community. This article reports changes to the taxonomy of viruses infecting archaea that were approved and ratified by the ICTV in March 2025. Six new families of head-tailed viruses expanded the order *Caudoviricetes* (realm *Duplodnaviria*); one new family of filamentous viruses was added to the order *Ligamenvirales* (realm *Adnaviria*); one new family of viruses with pleomorphic virions was included within a new phylum, new order and new class in the kingdom *Trapavirae* (realm *Monodnaviria*); finally, three new families were created for spindle-shaped viruses that remain unassigned to higher level taxa. The 25 new species represent viruses infecting a broad range of archaea, including members of the classes Archaeoglobi, Bathyarchaeia, Methanobacteria, Methanomicrobia, Nitrososphaeria and Poseidoniia. Most of these viruses have been discovered by metagenomics in samples derived from diverse environments, including ambient and extreme marine ecosystems, the gastrointestinal tract of humans and animals, anaerobic digesters and terrestrial hot springs. Following this taxonomic update, archaeal viruses are officially classified into a total of 163 virus species in 94 genera within 62 families.

## Introduction

Archaeal viruses comprise the least explored part of the global virosphere [1]. Of the 14,690 virus species established until 2025, only 138, i.e. <1 %, represented viruses infecting archaea [2]. Nevertheless, these viruses display remarkable diversity of virion structure and gene content, which distinguish them from viruses of bacteria and eukaryotes [3,6]. Historically, archaeal viruses have been primarily isolated from geothermal and hypersaline habitats, with only a handful of viruses from ambient ecosystems. Thus, the officially classified archaeal virosphere has been biased towards viruses of thermophilic and halophilic archaea. However, advances in metagenomics and the decision of the International Committee on Taxonomy of Viruses (ICTV) to classify viruses based on their genome sequence alone, without the need for isolation and demonstration of infectivity [7,8], have enabled the classification of viruses from more diverse environments and infecting hosts that cannot be (easily) cultivated under laboratory conditions.

The ICTV Archaeal Viruses Subcommittee oversees the advancement of taxonomy of viruses infecting archaea and currently includes 11 Study Groups [9]. During 2024, seven taxonomic proposals for the creation of 25 new species within 17 new genera and 11 new families were submitted to the ICTV Archaeal Viruses Subcommittee. All submitted proposals were accepted by the ICTV Executive Committee and subsequently ratified by the entire ICTV membership in March 2025.

The 25 new species represent viruses infecting a broad range of archaea from diverse environments, including ambient and extreme marine ecosystems, the gastrointestinal tract of humans and animals, anaerobic digesters and terrestrial hot springs. Head-tailed viruses, predicted to infect Marine Group II Archaea (order Poseidoniales), have been classified into three new families, expanding the recently created order *Magrovirales* [10] and adding a new order, *Adrikavirales*, to the class *Caudoviricetes* [11]. Ten spindle-shaped viruses distantly related to members of the family *Thaspiviridae* [12] and predicted to infect ammonia-oxidizing archaea (order Nitrososphaerales) were classified into the new family *Nipumfusiviridae* [13]. Non-lytic viruses with enveloped pleomorphic virions associated with the archaea of the class Archaeoglobi, a group of hyperthermophilic archaea inhabiting deep-sea hydrothermal vents, were classified into the family *Thalassapleoviridae*, within a new order *Ageovirales*, new class *Caminiviricetes* and new phylum *Calorviricota* [14]. Diverse viruses associated with Bathyarchaeia, a group of archaea common and abundant in sedimentary ecosystems, were classified into four new families [15]. Head-tailed bathyarchaeal viruses were classified into the families *Fuxiviridae* and *Kunpengviridae*; filamentous viruses into *Chiyouviridae*, a new family within the order *Ligamenvirales* of the realm *Adnaviria* [16]; and spindle-shaped viruses into the family *Huangdiviridae*. Head-tailed viruses infecting *Methanobrevibacter* species, the dominant methanogenic archaea in the gut of humans and other animals, were classified into the family *Usuviridae* [17], further expanding the order *Methanobavirales* [18,19] within the class *Caudoviricetes*. Finally, a spindle-shaped virus associated with methanogenic archaea of the genus *Methanosarcina* detected in anaerobic digestion batch microcosms was classified into the family *Eurekaviridae*. All species were named using a binomial format (genus name+species epithet), as mandated by the ICTV [20]. Except for two viruses, namely, the usuvirid Methanobrevibacter tailed virus 1 [17] and the thalassapleovirid Archaeoglobus veneficus pleomorphic virus 1 [14], which have been isolated, all other newly classified archaeal viruses were discovered by metagenomics, reflecting the general trend in current virus discovery efforts.

Following this taxonomic update, archaeal viruses are officially classified into a total of 163 virus species in 94 genera within 62 families. The individual summaries of the seven taxonomic proposals are described in more detail in the following section, and the full proposals can be accessed via the ICTV website (ictv.global). A file including all the Tables of taxonomic changes below is available as a supplementary file to this article.

## Main Text

## Contents

2024.001A.Apasviridae_newfam
2024.002A.Adrikavirales_neworder_2newfam
2024.003A.Nipumfusiviridae_newfam
2024.004A.Thalassapleoviridae_newphylum
2024.005A.Bathyarchaeia_4newfam
2024.006A.Usuviridae_newfam
2024.007A.Eurekaviridae_newfam

## 2024.001A.Apasviridae_newfam

**Title:** Create one new family in the order *Magrovirales* (class *Caudoviricetes*)

**Authors:** Prabhu A (apoorva.prabhu@uq.edu.au), Rinke C

**Summary**

**Taxonomic rank(s) affected**
Magrovirus group A (order *Magrovirales*; class *Caudoviricetes*)
**Description of current taxonomy**
Recently, the order *Magrovirales* was created for viruses associated with Marine Group II Archaea (order Poseidoniales), belonging to the class *Caudoviricetes*. Within *Magrovirales,* the family *Aoguangviridae* includes the group ‘Magrovirus B’ [21].
**Proposed taxonomic change(s)**
Here, we propose creating the family *Apasviridae* for the group ‘Magrovirus A’, with one new genus *Agnivirus,* which includes the species *Agnivirus brisbanense*.
**Justification**
Most genome sequences of magroviruses belonging to group A have not been deposited into public databases, i.e. GenBank. Hence, we propose the classification of viruses based on the demarcation criteria previously established for classification of archaeal-tailed viruses infecting halophilic and methanogenic archaea.
**Submitted**: 24/07/2023; **Revised**: 07/10/2024

**Table 1** *Apasviridae*, three new taxa*

**Table:** 

Operation	Rank	New taxon name	Virus name	Exemplar
New taxon	Family	*Apasviridae*		
New taxon	Genus	*Agnivirus*		
New taxon	Species	*Agnivirus brisbanense*	magrovirus_A_01	OR863078

^*^Source/full text: https://ictv.global/proposals/2024.001A.Apasviridae_newfam.zip

## 2024.002A.Adrikavirales_neworder_2newfam

**Title:** Create one new family in the order *Magrovirales* (class *Caudoviricete*s) and one new order *Adrikavirales* within the class *Caudoviricetes*

**Authors:** Prabhu A (apoorva.prabhu@uq.edu.au), Rinke C

**Summary**

**Taxonomic rank(s) affected**
Magrovirus group E (unofficially assigned to the order *Magrovirales;* class *Caudoviricetes*) and a new order within the class *Caudoviricetes*.
**Description of current taxonomy**
Recently, the order *Magrovirales* was created for viruses associated with Marine Group II Archaea (order Poseidoniales), belonging to the class *Caudoviricetes*. Within *Magrovirales,* the family *Aoguangviridae* includes the group ‘Magrovirus B’ [21].
**Proposed taxonomic change(s)**
Here, we propose creating the family *Krittikaviridae,* representing the group ‘Magrovirus E’, with one new genus *Velanvirus,* which will include the species *Velanvirus brisbanense*. In addition, we identified a virus associated with Poseidoniales, which belongs to a novel order (*Adrikavirales*), family (*Satyavativiridae*), genus (*Vyasavirus*) and species (*Vyasavirus brisbanense*) within the class *Caudoviricetes*.
**Justification**
Most currently available genomes of magroviruses assigned to group E are not of high quality and do not have GenBank entries. Furthermore, Poseidoniales-associated viruses assigned to an order other than *Magrovirales* have not been described. Hence, we propose the classification of viruses based on the demarcation criteria previously established for classification of archaeal-tailed viruses infecting halophilic and methanogenic archaea.
**Submitted**: 18/03/2024; **Revised**: 07/10/2024

**Table 2** Seven new taxa within the class Caudoviricetes*

**Table:** 

Operation	Rank	New taxon name	Virus name	Exemplar
New taxon	Family	*Krittikaviridae*		
New taxon	Genus	*Velanvirus*		
New taxon	Species	*Velanvirus brisbanense*	magrovirus_E_01	PP497039
New taxon	Order	*Adrikavirales*		
New taxon	Family	*Satyavativiridae*		
New taxon	Genus	*Vyasavirus*		
New taxon	Species	*Vyasavirus brisbanense*	Poseidoniales virus P01	PP497040

^*^Source/full text: https://ictv.global/proposals/2024.002A.Adrikavirales_neworder_2newfam.zip

## 2024.003A.Nipumfusiviridae_newfam

**Title:** Create one new family *Nipumfusiviridae* with four genera and ten species for archaeal viruses

**Authors:** Yimin Ni (Nemo.ni@outlook.com), Tianqi Xu, Shuling Yan, Lanming Chen, Yongjie Wang

**Summary**

**Taxonomic rank(s) affected**
We propose creating a new family, *Nipumfusiviridae*, for classification of spindle-shaped viruses infecting ammonia-oxidizing archaea. The family is not currently assigned to any higher level taxon.
**Description of current taxonomy**
Three families of small spindle-shaped archaeal viruses are currently defined: *Fuselloviridae, Halspiviridae* and *Thaspiviridae*. In addition, several spindle-shaped viruses are still unclassified. No spindle-shaped viruses infecting a methanogen have been classified so far.
**Proposed taxonomic change(s)**
We propose a new family for Nitrosopumilaceae virus NYM1 and its relatives, the *Nipumfusiviridae* (‘Ni’ and ‘pum’ for having sequence features similar to archaea from the family Nitrosopumilaceae and for being the deduced host; ‘fusi’ after the Latin word meaning spindles for the possible morphology). The four proposed genera are named *Yangshanfusivirus*, *Terrafusivirus*, *Marefusivirus* and *Baiafusivirus* after their original sampling sites, and species names are given based on the sampling locations.
**Justification**
Members of the *Nipumfusiviridae* are distantly related to members of the family *Thaspiviridae*. To be classified within *Nipumfusiviridae*, new members should share at least 30% average amino acid identity with the genomes of other viruses classified within the family *Nipumfusiviridae* and share a minimum set of homologous proteins, including the major capsid protein and the ATPase.
**Submitted***:* 20/05/2024

**Table 3** *Nipumfusiviridae*, 15 new taxa*

**Table:** 

Operation	Rank	New taxon name	Virus name	Exemplar
New taxon	Family	*Nipumfusiviridae*		
New taxon	Genus	*Marefusivirus*		
New taxon	Species	*Marefusivirus pacificense*	Nitrosopumilaceae spindle-shaped virus NMP1	BK067782
New taxon	Species	*Marefusivirus helgoense*	Nitrosopumilaceae spindle-shaped virus NMH1	BK067784
New taxon	Species	*Marefusivirus jervisense*	Nitrosopumilaceae spindle-shaped virus NMJ1	BK067785
New taxon	Species	*Marefusivirus columbiaense*	Nitrosopumilaceae spindle-shaped virus NMC1	BK067789
New taxon	Species	*Marefusivirus montereyense*	Nitrosopumilaceae spindle-shaped virus NMM1	BK067790
New taxon	Genus	*Terrafusivirus*		
New taxon	Species	*Terrafusivirus michiganense*	Nitrosopumilaceae spindle-shaped virus NTM1	BK067788
New taxon	Species	*Terrafusivirus tennesseense*	Nitrosopumilaceae spindle-shaped virus NTT1	BK067791
New taxon	Genus	*Baiafusivirus*		
New taxon	Species	*Baiafusivirus delawarense*	Nitrosopumilaceae spindle-shaped virus NBD1	BK067787
New taxon	Species	*Baiafusivirus chesapeakense*	Nitrosopumilaceae spindle-shaped virus NBC1	BK067786
New taxon	Genus	*Yangshanfusivirus*		
New taxon	Species	*Yangshanfusivirus mimetica*	Nitrosopumilaceae spindle-shaped virus NYM1	BK067792

^*^Source/full text: https://ictv.global/proposals/2024.003A.Nipumfusiviridae_newfam.zip

## 2024.004A.Thalassapleoviridae_newphylum

**Title:** Create a phylum within kingdom *Trapavirae* (realm *Monodnaviria*) for classification of hyperthermophilic archaeal viruses with pleomorphic virions

**Authors:** Baquero DP, Bignon EA, Krupovic M (mart.krupovic@pasteur.fr)

**Summary**

**Taxonomic rank(s) affected**
*Monodnaviria, Trapavirae*
**Description of current taxonomy**
The monodnavirian kingdom *Trapavirae* currently comprises a single family, *Pleolipoviridae,* which includes haloarchaeal viruses with enveloped pleomorphic virions and ssDNA or dsDNA genomes.
**Proposed taxonomic change(s)**
Here, we propose to classify viruses infecting hyperthermophilic marine archaea, distantly related to pleolipovirids, into a new family, *Thalassapleoviridae*, and include it in a new phylum within the kingdom *Trapavirae*.
**Justification**
Whole-genome phylogenomic analysis and maximum likelihood phylogenetic analysis based on the membrane fusion protein characteristic of members of the kingdom *Trapavirae* show that members of the proposed family *Thalassapleoviridae* form a monophyletic group separate from the haloarchaeal pleolipovirids and currently unclassified related viruses of methanogenic archaea.
**Submitted***:* 21/06/2024

**Table 4** *Thalassapleoviridae*, 12 new taxa*

**Table:** 

Operation	Rank	New taxon name	Virus name	Exemplar
New taxon	Phylum	*Calorviricota*		
New taxon	Class	*Caminiviricetes*		
New taxon	Order	*Ageovirales*		
New taxon	Family	*Thalassapleoviridae*		
New taxon	Genus	*Avenivirus*		
New taxon	Genus	*Aprofuvirus*		
New taxon	Genus	*Geogavirus*		
New taxon	Species	*Avenivirus atlanticense*	Archaeoglobus veneficus pleomorphic virus 1	BK065155
New taxon	Species	*Aprofuvirus guaymasense*	Archaeoglobus profundus pleomorphic virus 1	BK065154
New taxon	Species	*Geogavirus atlanticense*	Geoglobus acetivorans pleomorphic virus 1	BK065156
New taxon	Species	*Geogavirus guaymasense*	Geoglobus ahangari pleomorphic virus 1	BK065157
New taxon	Species	*Geogavirus pacificense*	Thalassapleovirus 2	BK065158

^*^Source/full text:https://ictv.global/proposals/2024.004A.Thalassapleoviridae_newphylum.zip

## 2024.005A.Bathyarchaeia_4newfam

**Title:** Create four new families for Bathyarchaeia viruses

**Authors:** Duan CH, Liu Y, Liu LR, Cai MW, Zhang R, Zeng QL, Koonin V E, Krupovic M, Li M (limeng848@szu.edu.cn)

**Summary**

Bathyarchaeia is an archaeal class widespread in marine and freshwater sediments. Here, we propose four new families for viruses identified by metagenomics and associated with hosts of the Bathyarchaeia class. The families *Fuxiviridae* and *Kunpengviridae* include head-tailed viruses of the class *Caudoviricetes* in the realm *Duplodnaviria*. The family *Chiyouviridae* includes filamentous viruses of the archaea-specific realm *Adnaviria*. The fourth putative family, *Huangdiviridae*, with only one representative genome, includes an archaea-specific spindle-shaped virus; the spindle-shaped viruses have not yet been classified at higher taxonomy ranks.

**Submitted**: 20/06/2024; **Revised**: 04/09/2024

**Table 5** Bathyarchaeia, 12 new taxa*

**Table:** 

Operation	Rank	New taxon name	Virus name	Exemplar
New taxon	Family	*Fuxiviridae*		
New taxon	Family	*Kunpengviridae*		
New taxon	Family	*Chiyouviridae*		
New taxon	Family	*Huangdiviridae*		
New taxon	Genus	*Taijivirus*		
New taxon	Genus	*Dafengvirus*		
New taxon	Genus	*Wargodvirus*		
New taxon	Genus	*Xuanyuanvirus*		
New taxon	Species	*Taijivirus yinyang*	Bathyarchaeia bifangarchaeales fuxivirus 1	PP467601
New taxon	Species	*Dafengvirus linsing*	Bathyarchaeia jinwuousiales Kupengvirus 1	PP467599
New taxon	Species	*Wargodvirus xiongnu*	Bathyarchaeia bifangarchaeales chiyouvirus 1	PP467602
New taxon	Species	*Xuanyuanvirus yandi*	Bathyarchaeia baizomonadales Huangdivirus 1	QMYA01000001

^*^Source/full text: https://ictv.global/proposals/2024.005A.Bathyarchaeia_4newfam.zip

## 2024.006A.Usuviridae_newfam

**Title:** Create new family, *Usuviridae*, with two genera in the order *Methanobavirales* (class *Caudoviricetes*)

**Authors:** Diana P. Baquero, Sofia Medvedeva, Guillaume Borrel, Simonetta Gribaldo, Mart Krupovic (mart.krupovic@pasteur.fr)

**Summary**

**Taxonomic rank(s) affected**
*Duplodnaviria*, *Heunggongvirae*, *Uroviricota*, *Caudoviricetes*, *Methanobavirales*
**Description of current taxonomy**
The order *Methanobavirales* (class *Caudoviricetes*) currently includes five families of viruses infecting methanogenic archaea.
**Proposed taxonomic change(s)**
Create a new family, *Usuviridae,* with two genera for classification of viruses infecting human- and animal-gut-associated methanogenic archaea, and include this family in the existing order *Methanobavirales*.
**Justification**
Whole-proteome-based phylogenomic analysis using VipTree placed Methanobrevibacter smithii tailed virus 1-like viruses in a distinct clade, outside of the recently established families of tailed viruses associated with methanogenic archaea or other archaeal hosts.
**Submitted**: 21/06/2024; **Revised**: 11/09/2024

**Table 6** *Usuviridae*, five new taxa*

**Table:** 

Operation	Rank	New taxon name	Virus name	Exemplar
New taxon	Family	*Usuviridae*		
New taxon	Genus	*Manusuvirus*		
New taxon	Genus	*Hewusuvirus*		
New taxon	Species	*Manusuvirus methanobrevibacteri*	Methanobrevibacter smithii tailed virus 1	PP537965
New taxon	Species	*Hewusuvirus methanobrevibacteri*	Methanobrevibacter gottschalkii virus vir075	BK068243

^*^Source/full text: https://ictv.global/proposals/2024.006A.Usuviridae_newfam.zip

## 2024.007A.Eurekaviridae_newfam

**Title:** Create a new family, *Eurekaviridae*, of spindle-shaped archaeal virus

**Authors:** Coves M, Krupovic M, Bize A (ariane.bize@inrae.fr)

**Summary**

**Taxonomic rank(s) affected**
We suggest creating a new family, a new genus and a new species for classification of a spindle-shaped archaeal virus predicted to infect *Methanosarcina* species.
**Description of current taxonomy**
Three families of small spindle-shaped archaeal viruses are currently defined: *Fuselloviridae, Halspiviridae* and *Thaspiviridae*. In addition, several spindle-shaped viruses are still unclassified. No spindle-shaped viruses infecting methanogenic archaea have been classified so far.
**Proposed taxonomic change(s)**
We suggest creating a new family (*Eurekaviridae*), a new genus (*Hesperidvirus*) and a new species (*Hesperidvirus aureum*) to classify a newly sequenced uncultured virus, Methanosarcina spindle-shaped virus 1.
**Justification**
The complete, circular Methanosarcina spindle-shaped virus 1 genome has been obtained through metavirome co-assembly, from samples collected in mesophilic anaerobic digestion batch microcosms fed with biowaste. This genome encodes two copies of the major coat protein similar to those of previously characterized spindle-shaped viruses. However, it does not show significant genomic similarity to other archaeal spindle-shaped viruses, which justifies the creation of a new family.
**Submitted**: 26/06/2024; **Revised**: 02/10/2024

**Table 7** *Eurekaviridae*, three new taxa*

**Table:** 

Operation	Rank	New taxon name	Virus name	Exemplar
New taxon	Family	*Eurekaviridae*		
New taxon	Genus	*Hesperidvirus*		
New taxon	Species	*Hesperidvirus aureum*	Methanosarcina spindle-shaped virus 1	PQ167755

^*^Source/full text: https://ictv.global/proposals/2024.007A.Eurekaviridae_newfam.zip

## Supplementary material

10.1099/jgv.0.002117 Uncited Table S1.

## Abbreviations

ICTV: International Committee on Taxonomy of Viruses
