# Erratum: Summary of taxonomy changes ratified by the International Committee on Taxonomy of Viruses (ICTV) from the Archaeal Viruses Subcommittee, 2025

**DOI:** 10.1099/jgv.0.002145

**Published:** 2025-08-14

**Authors:** Mart Krupovic, Diana P. Baquero, Eduardo A. Bignon, Ariane Bize, Guillaume Borrel, Mingwei Cai, Lanming Chen, Marion Coves, Changhai Duan, Simonetta Gribaldo, Eugene V. Koonin, Meng Li, Lirui Liu, Yang Liu, Ying Liu, Sofia Medvedeva, Yimin Ni, Apoorva Prabhu, Christian Rinke, Yongjie Wang, Tianqi Xu, Shuling Yan, Qinglu Zeng, Rui Zhang, ICTV Taxonomy Summary Consortium

**Affiliations:** 1 Institut Pasteur, Université Paris Cité, CNRS UMR6047, Archaeal Virology Unit, Paris, France; 2 Université Paris-Saclay, INRAE, PROSE, Antony, France; 3 Institut Pasteur, Université Paris Cité, CNRS UMR6047, Evolutionary Biology of the Microbial Cell, Paris, France; 4 Institute for Advanced Study, Shenzhen University, Shenzhen, PR China; 5 College of Food Science and Technology, Shanghai Ocean University, Shanghai, PR China;; 6 The Hong Kong University of Science and Technology, Hong Kong, PR China; 7 Computational Biology Branch, Division of Intramural Research, National Library of Medicine, National Institutes of Health, Bethesda, MD, USA; 8 Australian Centre for Ecogenomics, The University of Queensland, Brisbane, Australia; 9 University of Innsbruck, Innsbruck, Austria; 10 Entwicklungsgenetik und Zellbiologie der Tiere, Philipps-Universität Marburg, Marburg, Germany; 11 Department of Ocean Science, The Hong Kong University of Science and Technology, Hong Kong, PR China

In the published version of this article, there was an author omitted from the author list.

This was Ying Lui, Institut Pasteur, Université Paris Cité, CNRS UMR6047, Archaeal Virology Unit, Paris, France

Previously, the author and affiliation list appeared as below:

Mart Krupovic^1, *^, Diana P. Baquero^1^, Eduardo A. Bignon^1^, Ariane Bize^2^, Guillaume Borrel^3^, Mingwei Cai^4^, Lanming Chen^5^, Marion Coves^2^, Changhai Duan^4,6^, Simonetta Gribaldo^3^, Eugene V. Koonin^7^, Meng Li^4^, Lirui Liu^4^, Yang Liu^4^, Sofia Medvedeva^1^, Yimin Ni^5^, Apoorva Prabhu^8^, Christian Rinke^9^, Yongjie Wang^5^, Tianqi Xu^5^, Shuling Yan^10^, Qinglu Zeng^11^, Rui Zhang^4^ and ICTV Taxonomy Summary Consortium

Author affiliations:

^1^Institut Pasteur, Université Paris Cité, CNRS UMR6047, Archaeal Virology Unit, Paris, France; ^2^Université Paris-Saclay, INRAE, PROSE, Antony, France; ^3^Institut Pasteur, Université Paris Cité, CNRS UMR6047, Evolutionary Biology of the Microbial Cell, Paris, France; ^4^Institute for Advanced Study, Shenzhen University, Shenzhen, PR China; ^5^College of Food Science and Technology, Shanghai Ocean University, Shanghai, PR China; ^6^The Hong Kong University of Science and Technology, Hong Kong, PR China;^ 7^Computational Biology Branch, Division of Intramural Research, National Library of Medicine, National Institutes of Health, Bethesda, MD, USA; ^8^Australian Centre for Ecogenomics, The University of Queensland, Brisbane, Australia; ^9^University of Innsbruck, Innsbruck, Austria; ^10^Entwicklungsgenetik und Zellbiologie der Tiere, Philipps-Universität Marburg, Marburg, Germany; ^11^Department of Ocean Science, The Hong Kong University of Science and Technology, Hong Kong, PR China.

The correct author and affiliation list should have appeared as:

Mart Krupovic^1, *^, Diana P. Baquero^1^, Eduardo A. Bignon^1^, Ariane Bize^2^, Guillaume Borrel^3^, Mingwei Cai^4^, Lanming Chen^5^, Marion Coves^2^, Changhai Duan^4,6^, Simonetta Gribaldo^3^, Eugene V. Koonin^7^, Meng Li^4^, Lirui Liu^4^, Yang Liu^4^, Ying Liu^1^, Sofia Medvedeva^1^, Yimin Ni^5^, Apoorva Prabhu^8^, Christian Rinke^9^, Yongjie Wang^5^, Tianqi Xu^5^, Shuling Yan^10^, Qinglu Zeng^11^, Rui Zhang^4^ and ICTV Taxonomy Summary Consortium

Author affiliations:

^1^Institut Pasteur, Université Paris Cité, CNRS UMR6047,Archaeal Virology Unit, Paris, France; ^2^Université Paris-Saclay, INRAE, PROSE, Antony, France; ^3^Institut Pasteur, Université Paris Cité, CNRS UMR6047, Evolutionary Biology of the Microbial Cell, Paris, France; ^4^Institute for Advanced Study, Shenzhen University, Shenzhen, PR China; ^5^College of Food Science and Technology, Shanghai Ocean University, Shanghai, PR China; ^6^The Hong Kong University of Science and Technology, Hong Kong, PR China;^ 7^Computational Biology Branch, Division of Intramural Research, National Library of Medicine, National Institutes of Health, Bethesda, MD, USA; ^8^Australian Centre for Ecogenomics, The University of Queensland, Brisbane, Australia; ^9^University of Innsbruck, Innsbruck, Austria; ^10^Entwicklungsgenetik und Zellbiologie der Tiere, Philipps-Universität Marburg, Marburg, Germany; ^11^Department of Ocean Science, The Hong Kong University of Science and Technology, Hong Kong, PR China.

The version of record of this article will be updated to reflect the correction to the author list.

The Microbiology Society apologises for any inconvenience caused.

